# E2F1 mediates competition, proliferation and response to cisplatin in cohabitating resistant and sensitive ovarian cancer cells

**DOI:** 10.3389/fonc.2024.1304691

**Published:** 2024-01-26

**Authors:** Andres Valdivia, Matthew Cowan, Horacio Cardenas, Ana Maria Isac, Guangyuan Zhao, Hao Huang, Daniela Matei

**Affiliations:** ^1^ Department of Obstetrics and Gynecology, Feinberg School of Medicine, Northwestern University, Chicago, IL, United States; ^2^ Department of Obstetrics & Gynecology and Women’s Health, Montefiore Medical Center, Bronx, NY, United States; ^3^ Robert H. Lurie Comprehensive Cancer Center, Feinberg School of Medicine, Northwestern University, Chicago, IL, United States; ^4^ Department of Medicine, Jesse Brown Veterans Affairs Medical Center, Chicago, IL, United States

**Keywords:** chemotherapy, chemoresistance, ovarian cancer, cell proliferation, tumor heterogeneity

## Abstract

**Background:**

Tumor heterogeneity is one of the key factors leading to chemo-resistance relapse. It remains unknown how resistant cancer cells influence sensitive cells during cohabitation and growth within a heterogenous tumors. The goal of our study was to identify driving factors that mediate the interactions between resistant and sensitive cancer cells and to determine the effects of cohabitation on both phenotypes.

**Methods:**

We used isogenic ovarian cancer (OC) cell lines pairs, sensitive and resistant to platinum: OVCAR5 vs. OVCAR5 CisR and PE01 vs. PE04, respectively, to perform long term direct culture and to study the phenotypical changes of the interaction of these cells.

**Results:**

Long term direct co-culture of sensitive and resistant OC cells promoted proliferation (p < 0.001) of sensitive cells and increased the proportion of cells in the G1 and S cell cycle phase in both PE01 and OVCAR5 cells. Direct co-culture led to a decrease in the IC50 to platinum in the cisplatin-sensitive cells (5.92 µM to 2.79 µM for PE01, and from 2.05 µM to 1.51 µM for OVCAR5). RNAseq analysis of co-cultured cells showed enrichment of Cell Cycle Control, Cyclins and Cell Cycle Regulation pathways. The transcription factor E2F1 was predicted as the main effector responsible for the transcriptomic changes in sensitive cells. Western blot and qRT-PCR confirmed upregulation of E2F1 in co-cultured vs monoculture. Furthermore, an E2F1 inhibitor reverted the increase in proliferation rate induced by co-culture to baseline levels.

**Conclusion:**

Our data suggest that long term cohabitation of chemo-sensitive and -resistant cancer cells drive sensitive cells to a higher proliferative state, more responsive to platinum. Our results reveal an unexpected effect caused by direct interactions between cancer cells with different proliferative rates and levels of platinum resistance, modelling competition between cells in heterogeneous tumors.

## 1 Introduction

Among the principal discoveries that have shaped contemporary oncology, tumor heterogeneity emerges as a fundamental concept for understanding of the process cancer progression and development of chemotherapy resistance (1). Tumor heterogeneity refers to the presence of diverse cellular and genetic phenotypes within a tumor, principally due to clonal evolution and genetic diversity and posing substantial obstacles to cancer diagnosis, treatment, and prognosis (2). Tumors are composed of cancer cells, cancer-associated fibroblasts (CAFs), endothelial cells, monocytes, macrophages, dendritic cells, T-cells, lymphocytes, among others (3). Anti-tumor therapies can significantly alter the composition of cells in solid tumors, modifying the proportions of cells that form tumors and their interactions (4). During tumor development, therapy-sensitive and resistant cells develop and are in constant interaction with one another (5). It remains unknown whether and how drug-resistant tumor cells influence sensitive cells during these early conditions of cohabitation.

The E2F family has emerged as a critical player in controlling cell cycle progression, DNA replication, and apoptosis (6). Among the E2F family members, E2F1 was identified as a master regulator of cell cycle progression and is involved in DNA damage response and apoptosis (7). E2F1 interacts with the retinoblastoma protein (pRB), a key tumor suppressor that is frequently mutated in cancer and with other RB family proteins (RBL1 and RBL2) that modulate its transcriptional activity (8). The binding of E2F1 to pRB blocks its transactivation domain, preventing recruitment of co-activators to the promoters of target genes and transcription activation. E2F1 is involved in the regulation of genes crucial for cell cycle progression, including those involved in G1/S transition and DNA replication, thus governing the fine balance between cell proliferation and quiescence. Depending on the cellular context, E2F1 can also promote cell survival or induce apoptosis (9).

The aims of this project were to understand the driving factors mediating interactions between resistant and sensitive cancer cells and the effects of cohabitation on both cellular phenotypes. To accomplish this goal, we used a co-culture model allowing direct interaction between isogenic ovarian cancer cells with different levels of sensitivity to platinum and mimicking a diverse tumor cell population. Proliferation assays and transcriptomic analyses demonstrate that cohabitating cells with distinct proliferation rates promote cell competition, increase cell cycling, and responsiveness to chemotherapy.

## 2 Materials and methods

### 2.1 Cell lines, reagents, and antibodies

PE01 and PE04 cells were purchased from Sigma-Aldrich (#10032308-1VL and #10032309-1VL respectively). OVCAR5 cells were obtained from Dr. Marcus Peter at Northwestern University. OVCAR5 Cisplatin Resistant (CisR) cells were generated in our laboratory applying consecutive cisplatin treatments as described before (10). Cells were maintained at 37 °C in an environment of 5% CO_2_ and 100% humidity. All media used for maintaining the cell lines are included in **Supplementary Table S1**. Cells were confirmed to be pathogen and mycoplasma-free by Charles River Animal Diagnostic Services and used at low passages for all the experiments performed. Cell lines were authenticated by IDEXX BioAnalytics with short tandem repeat (STR) profiling. Palbociclib (#PZ0383-5MG) and HLM006474 (#SML1260-5MG) were obtained from Sigma Aldrich. A list of antibodies, primers and their sources are included in **Supplementary Tables S2** and **S3** respectively.

### 2.2 Cell transfection

GFP and RFP stable PE01, PE04 and OVCAR5 cells were generated by cell transduction with lentiviral vectors (Gentarget #LVP001 and #LVP023, respectively) followed by selection with puromycin and flow cytometry-based cell sorting. Cells stably expressing GFP or RFP were cultured in the same media as the parental cells (**Supplementary Table S1**). The new stable cell lines were labeled PE01 RFP, PE04 GFP, OVCAR5 GFP and OVCAR5 CisR RFP.

### 2.3 Fluorescence microscopy and image processing

Transfected cells and co-cultured cells were observed using fluorescence microscopy. Images were acquired using either a Carl Zeiss Axiovert 200 microscope, equipped with an AxioCam HRC and a HAL 100 halogen lamp, with the following settings: Green channel: emission wavelength 515 nm, excitation wavelength 450-490 nm; Red channel: emission wavelength 590 nm, excitation wavelength 546 nm. Alternatively, a Nikon A1 confocal microscope system was used with the following settings: Alexa Fluor 488: emission wavelength 525 nm, excitation wavelength 488 nm; Alexa Fluor 568: emission wavelength 595 nm, excitation wavelength 561 nm. For both microscope systems, image enhancement, including adjustments to brightness and contrast at every pixel, was carried out using Fiji software (ImageJ). Additionally, signal quantification (fluorescence area) was performed with the same software.

### 2.4 Cancer cells co-culture

For co-culture of PE01 RFP (platinum sensitive) with PE04 GFP (platinum resistant), and OVCAR5 GFP (platinum sensitive) with OVCAR5 CisR RFP (platinum resistant), different ratios of cells were used (Sensitive to Resistant; 1:1, 1:2, 1:5, 1:7). Cells were seeded on a 10cm dish and grown for up to 14 days. Co-cultures were evaluated every 3 days under a fluorescent microscope to assess their growth. On day 14, cells were trypsinized and separated using a FACS sorter (FACSMelody, BD Bioscience) for further analyses.

### 2.5 Cell proliferation assay

Cell proliferation at each time point was estimated by using CCK8 (APExBIO #K1018) following the manufacturer’s protocol. Absorbance (450nM) was measured with a microplate reader (BioTek ELX800). Quantification of cell proliferation was estimated using GraphPad Prism 6 using day 1 as the starting point.

### 2.6 Half maximal inhibitory concentration determinations

The IC_50_ values of the different treatments and chemical were determined using CCK8 kit as described before (10). IC_50_ values were determined by logarithm-normalized sigmoidal dose curves fitting using GraphPad Prism 6.

### 2.7 RNA extraction and qRT-PCR analysis

RNA was isolated by using TRIzol (Invitrogen) according to the manufacturer’s instructions and quantified using a NanoDrop 3300 Fluorospectrometer. mRNA was generated using 1 μg RNA and reverse transcribed into cDNA using the iScript cDNA synthesis kit (Bio-Rad) and manufacturer’s protocol. qPCR analysis was performed by using iTaq Universal SYBR green Supermix (Bio-Rad) and an AB 7900HT instrument (Applied Biosystems). 18S RNA was used as endogenous control. The primer sequences for E2F1 and GAPDH are included in **Supplementary Table S3**. PCR was performed using the following parameters: 94°C for 10 minutes, 40 cycles of amplification at 94°C for 15 seconds and 60°C for 1 minute, followed by an extension step of 7 minutes at 72°C. The ΔΔCt method was used to calculate relative expression of target genes. Results are presented as means ± SD. Experiments were performed in triplicate.

### 2.8 RNA sequencing and pathway analysis

The RNA sequencing (RNA-seq) libraries were prepared from 1 μg of total RNA of PE01 RFP, and PE04 GFP. Libraries were generated using the NEBNext Ultra II RNA library prep kit from Illumina (New England Biolabs, Inc.). Library preparation, sequencing, and analysis was performed according to previous publications (11, 12). FDR correction was applied for multiple hypothesis testing. Pathway and regulator analyses were performed using Metascape (13) and Ingenuity Pathway Analysis (IPA) (Qiagen).

### 2.9 Western blotting

Proteins were extracted using RIPA buffer and quantification was performed using the Bradford assay. Proteins were resolved by PAGE and then transferred onto a PVDF membrane using a wet electroblotting system. Membranes were incubated in TBS-Tween 5% BSA for one hour (blocking) and incubated with primary antibodies (1:1000 dilution, overnight at 4°C). After incubation with the secondary antibody (anti rabbit/mouse-horseradish peroxidase 1:1000 dilution) for 1 h at room temperature, signal was developed using SuperSignal West Pico PLUS Chemiluminescent Substrate (Thermo Fisher Scientific cat#: 34580) and captured with an ImageQuant LAS 4000 machine. To detect additional proteins, membranes were treated with Restore Western Blot Stripping Buffer (Thermo Fisher Scientific cat#: 21059), blocked, and then incubated with primary antibody. Western blots were quantified using Fiji software (ImageJ).

### 2.10 Flow cytometry and cell cycle analysis

After co-culture at the ratios indicated above, cells were trypsinized, and then resuspended in 2% BSA in PBS for sorting on a BD FACSMelody (BD Bioscience). Cell sorter with fluorescein isothiocyanate (FITC) channel for GFP and PE-YG for RFP. For evaluation of cell cycle, cells were stained with propidium iodide (PI) and run on a LSRFortessa Cell Analyzer (BD Bioscience). Data were analyzed and quantified using Flowjo v10.8.1 (BD Bioscience).

### 2.11 Statistical analysis

Data are presented as means ± SD with associated p-values. Statistical analysis was performed by using two-tailed Student’s t test, one-way ANOVA, or two-way ANOVA (Prism - GraphPad Software). P values <0.05 were considered statistically significant.

## 3 Results

### 3.1 Co-culture of sensitive and platinum-resistant cells increases proliferation and platinum-sensitivity of sensitive cells

**Figure 1:**
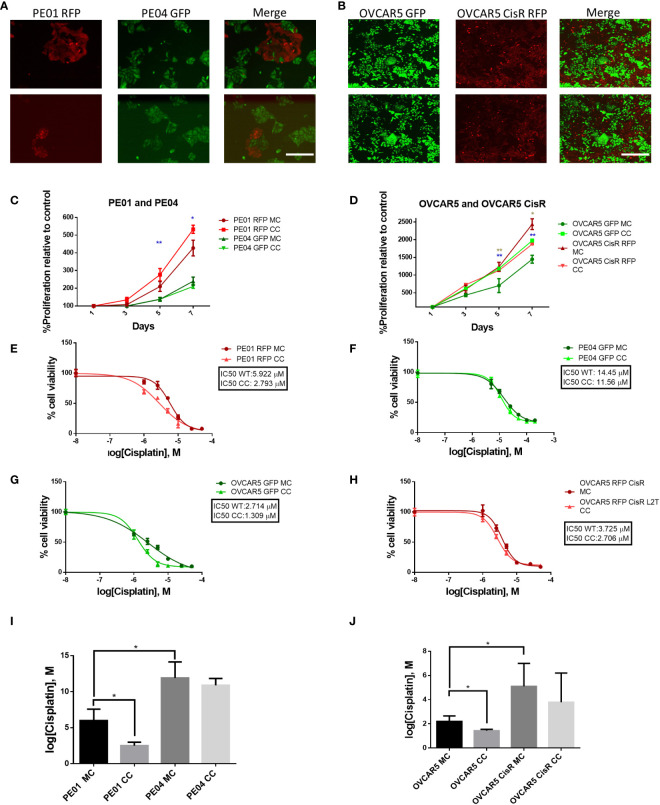
Long term co-culture of platinum-sensitive and resistant cells increases proliferation and sensitivity to platinum of sensitive cells. **(A, B)** Pictures of co-cultured PE01 and PE04 **(A)** and OVCAR5 WT and OVCAR5 CisR **(B)** ovarian cancer cells transduced with RFP or GFP lentiviral vectors. Images show cells at day 5 of co-culture at the ratios of 1:5 (sensitive to resistant) (2 replicates each, scale bar: 200 µm). **(C, D)** Cell proliferation (mean ± SD, n=3) measured with CCK8 assays in PE01 and PE04 **(C)** and OVCAR5 WT and CisR **(D)** cells maintained in co-culture (CC) or monoculture (MC). Cells were co-cultured for 14 days, sorted by FACS and then cultured to evaluate cell viability. Blue asterisk indicates statistical significance for the comparison between sensitive cells, marron asterisk indicates statistical significance for the comparison between resistant cells **(E-H)** Representative curves of cisplatin effects on cell viability of PE01 **(E)**, PE04 **(F)**, OVCAR5 WT **(G)** and OVCAR5 CisR **(H)** maintained in co-culture as described in **(A, B)** vs. the same cell lines in monoculture. **(I, J)** Comparison of cisplatin IC_50_ values (means ± SD, n=3) between monocultured and co-cultured PE01 with PE04 **(I)** and OVCAR5 WT with CisR cells **(J)**. All graphs are representative of 3 independent replicates. *p<0.05; **p<0.01.

We used two isogenic sets of OC cells lines with different levels of sensitivity to platinum (i.e., PE01 vs. PE04 and OVCAR5 (parental) vs. OVCAR5 CisR) and labeled with fluorescent markers (GFP or RFP) to perform direct and indirect co-culture experiments to determine if co-culture impacts their phenotype. PE01 and PE04 cells are derived from the same patient diagnosed with high grade serous OC at the time of a platinum sensitivity (PE01, cisplatin IC50 = 4.7µM) or resistant recurrence (PE04, cisplatin IC50 = 14.0µM) (14). OVCAR5 CisR (IC50 = 4.05µM) was derived from OVCAR5 cells (IC50 = 1.85µM) after repeated exposures to cisplatin, as previously described (10). Both paired cell lines were able to grow together in long term co-culture when seeded at different ratios. Here we chose a 1:5 ratio of sensitive to resistant cells, respectively as the optimal condition for further experiments. Representative images of the co-cultures, taken on day 5 are shown in **Figures 1A, B**. After 14 days of direct co-culture, cells were separated using FACS based on GFP or RFP expression and proliferation rates were measured by using a CCK8 colorimetric assay. Both PE01 and parental OVCAR5 cells separated after direct co-culture proliferated significantly faster compared to cells grown as monoculture (**Figures 1C**, p < 0.01 and 1D, p < 0.001). In contrast, only a slight change in proliferation was observed for the resistant cells, PE04 and OVCAR5-CisR (**Figures 1C, D**). Interestingly, this effect on cell proliferation was not observed when monolayer PE01 and PE04 cells were treated with conditioned media (CM) from PE04 and PE01 cells respectively (indirect co-culture, **Supplementary Figure S1A**), suggesting that direct contact or close proximity between cells in co-culture is a key determinant of the observed effects on cell growth rates.

Next, we tested whether co-culture of isogenic cells altered response to cisplatin treatment. We observed increased sensitivity of the sensitive cells after 14 days of co-culture with cisplatin resistant cells; for instance, the cisplatin IC_50_ of PE01 cells was reduced from 5.922 µM (monoculture) to 2.793 µM (co-culture, **Figures 1E, I**, p < 0.05), whilst for OVCAR5 cells, the cisplatin IC_50_ went from 2.054 µM (monoculture) to 1.516 µM (co-culture) (**Figures 1G, J**, p < 0.05). The same trend was observed in platinum resistant PE04 (**Figures 1F, I**) and OVCAR5 CisR cells (**Figure 1H, J**), however the changes were not statistically significant for resistant cells (**Figure 1I, J**). Changes to platinum IC_50_ were not observed when PE01 and PE04 cells were treated with CM from the paired cell line (indirect co-culture, **Supplementary Figures S1B, C**).

### 3.2 Competition of sensitive and resistant cells in co-culture promotes the proliferation of the sensitive cells

**Figure 2:**
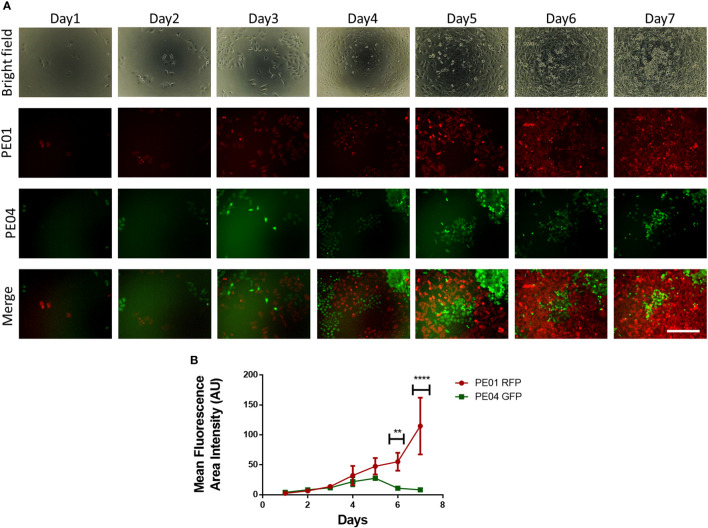
Long term co-culture of platinum-sensitive and resistant cancer cells induces cell competition. **(A)** Representative fluorescence images of co-cultured PE01 and PE04 cells. Cells were seeded in 96-wells at a ratio of 1:2 (PE01:PE04) and co-cultured for 7 days. Scale bar = 200 µm). **(B)** Quantification plot of cell numbers estimated using fluorescence density of the cultures described in **(A)** (n=3; **p<0.01; ****p<0.0001; comparing PE01 vs. PE04 proliferation rates at different timepoints).

To study the dynamic between proliferating sensitive and resistant cancer cells, we followed the numbers of cohabitating cells by using fluorescence microscopy over a period of 7 days. Seeded at a ratio of 1:2, PE01 cells started at a lower concentration compared to the PE04 cells (**Figure 2A**); however, after day 4, PE01 cells began to multiply and take over the co-culture, reaching higher numbers on day 6 and a peak on day 7 (**Figure 2B**). On the other hand, PE04 cells proliferated seemingly at similar rates during the first 3 days. PE04 cell numbers were significantly reduced after day 4, followed by a maximal reduction reached at day 7 (**Figure 2B**). These results indicate that PE01 and PE04 compete for the limited space and resources present under co-culture conditions. This competition in turn increased the proliferation rate of the sensitives cells (as observed in **Figure 1C**) resulting in the overtaking of the dish by PE01 cells and suppression of PE04 cells’ growth.

### 3.3 RNA sequencing analysis of co-cultured PE01 cells shows enrichment of cell cycle related genes

To elucidate the mechanism responsible for the observed increased proliferation of sensitive cells during the co-culture with isogenic platinum resistant cells, we performed RNA sequencing of PE01 cells maintained in monoculture, PE01 cells co-cultured with PE04, and PE01 cells treated with CM of PE04 cells for 14 days. Analysis of RNA sequencing data detected 3840 differentially expressed protein-coding genes (DEGs) of which 1774 were upregulated and 2066 downregulated in sensitive PE01 cells co-cultured with PE04 cells compared with PE01 cells in monoculture (FDR< 0.05; top DEGs included in **Supplementary Table S4**). RNA sequencing analysis of PE01 cells treated with CM from PE04 cells vs. PE01 monoculture cells detected 1237 DEGs; 635 upregulated and 602 downregulated genes in PE01 cells treated with CM (FDR< 0.05; top DEGs included in **Supplementary Table S5**).

**Figure 3:**
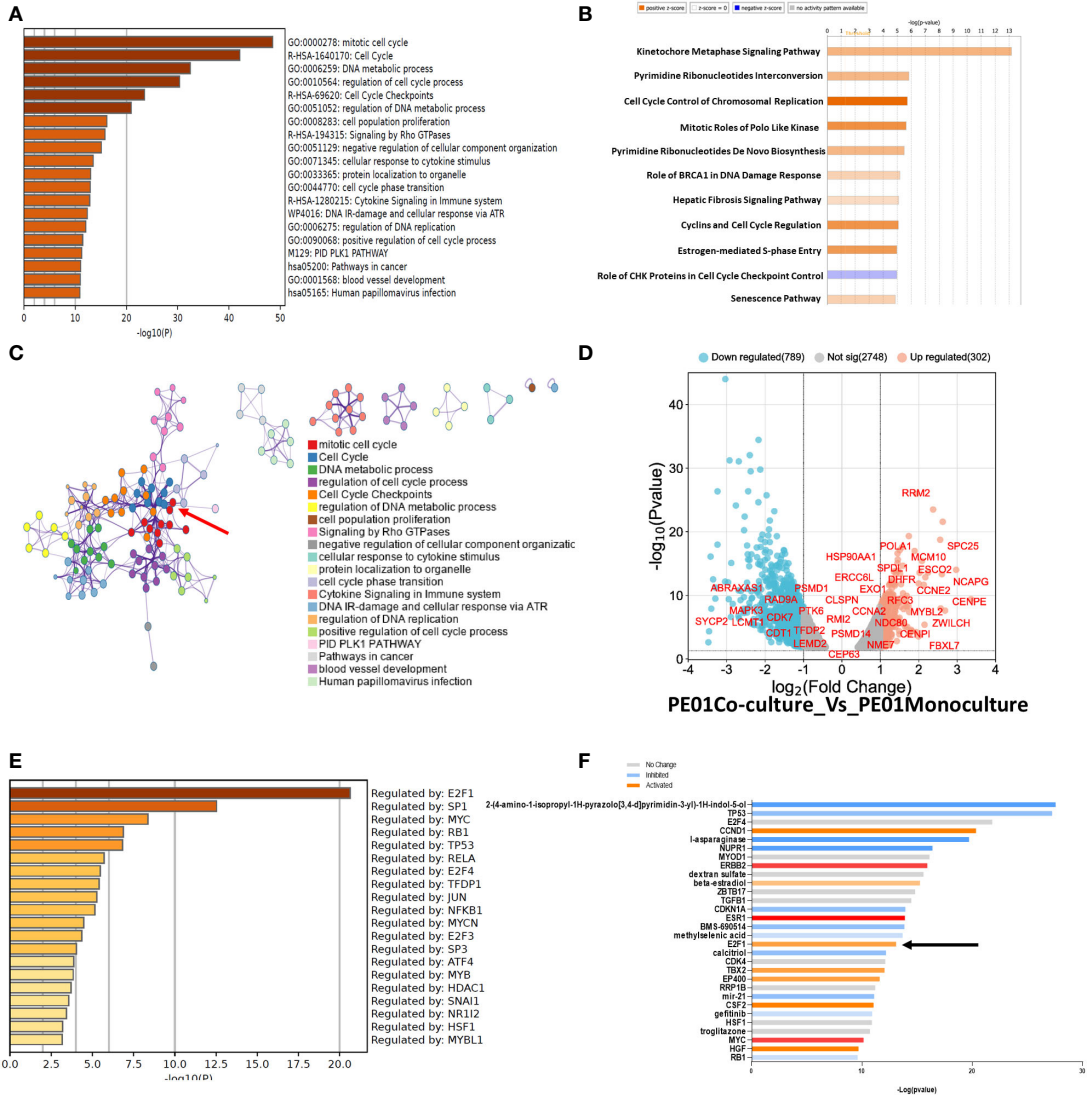
RNAseq of co-cultured cells promotes enrichment in genes related to cell cycle. **(A)** Pathway Enrichment analysis (Metascape) compares DEGs (FDR<0.05) determined by RNAseq between co- vs. monocultured PE01 with PE04 cells. Cells were co-cultured for 14 days and then selected by FACS for RNAseq. **(B)** Pathway Enrichment analysis performed with IPA of DEGs between co- and monocultured PE01 with PE04 cells. **(C)** Protein-protein interaction analysis between co- and monocultured PE01 cells (Metascape) shows a highly defined cluster of proteins related to cell cycle pathways (red arrow). **(D)** Volcano plot of the RNAseq data of co- and monocultured PE01 cells showing an upregulation of cell-cycle regulation genes. **(E)** Main regulator analysis (Metascape) shows E2F1 as the main regulator of cell cycle enriched pathways. **(F)** Gene activation analysis (IPA) shows increased activation of Cell Cycle related proteins, including E2F1 (black arrow).

Next, we performed pathway enrichment analysis of DEGs with a fold change of 1.5 or higher and 2-fold lower, using Metascape and Ingenuity (IPA). Metascape showed an enrichment of pathways related to *Mitotic cell cycle, Cell cycle*, and *DNA metabolic process* among other pathways related to cell cycle in PE01 co-cultured with PE04 cells vs PE01 cells maintained as monoculture (**Figure 3A**). Similarly, IPA analysis showed enrichment of cell cycle related pathways (*Cell Cycle Control of Chromosomal Replication, Cyclins and Cell Cycle Regulation*, among others; **Figure 3B**) in the co-cultured PE01 cells. *Protein-to-protein interaction* analysis performed using Metascape identified a core of proteins related to cell cycle regulation in the interaction matrix among DEGs from PE01 cells co-cultured with PE04 (**Figure 3C**, red arrow). In addition, a volcano plot of genes related to cell cycle regulation showed upregulation of genes involved in cell-cycle progression and G1-S phase in the co-cultured PE01 cells (**Figure 3D**). Further examination of the data using Metascape *Main Regulator Analysis* identified the transcription factor E2F1 as the top regulator among the upregulated genes in PE01 cells co-cultured with PE04 cells vs PE01 cells cultured alone (**Figure 3E**). IPA also predicted E2F1 as one of the key regulators among DEGs in co-cultured PE01 cells (**Figure 3F**). In contrast, analysis using Metascape of DEGs in PE01 cells treated with CM of PE04 cells vs. PE01 cells cultured alone showed no enrichment in cell cycle related genes (**Supplementary Figure S2A**). These differences were evident when comparing the enriched pathways in each condition; in the case of the direct co-culture, pathways related with cell cycle control and progression were enriched (black arrows) while in the case of indirect co-culture, VEGF-VEGFR, cytokine and interferon signaling were observed (**Supplementary Figure S2B**). Metascape analysis of main regulators for differentially expressed genes from PE01 cells treated with CM of PE04 cells predicted that STAT3, JUND, SP1, but not E2F1, were the main regulators of transcriptomic changes (**Supplementary Figure S2C**). Furthermore, when comparing the main regulators of both conditions, activation of E2F family genes like E2F1, E2F3 and E2F4 were only found in the analysis of PE01 co-cultured with PE04 cells and not in the comparison between PE01 cultured alone vs. with PE04 CM (**Supplementary Figure S2D**, black arrows). Taken together, these results indicate that the direct co-culture of sensitive and resistant cells led to activation of E2F family of transcription factors and upregulation of cell cycle related genes in the cisplatin sensitive cells.

### 3.4 Co-culture of sensitive and resistant cells promote cell cycle progression of sensitive cells

**Figure 4:**
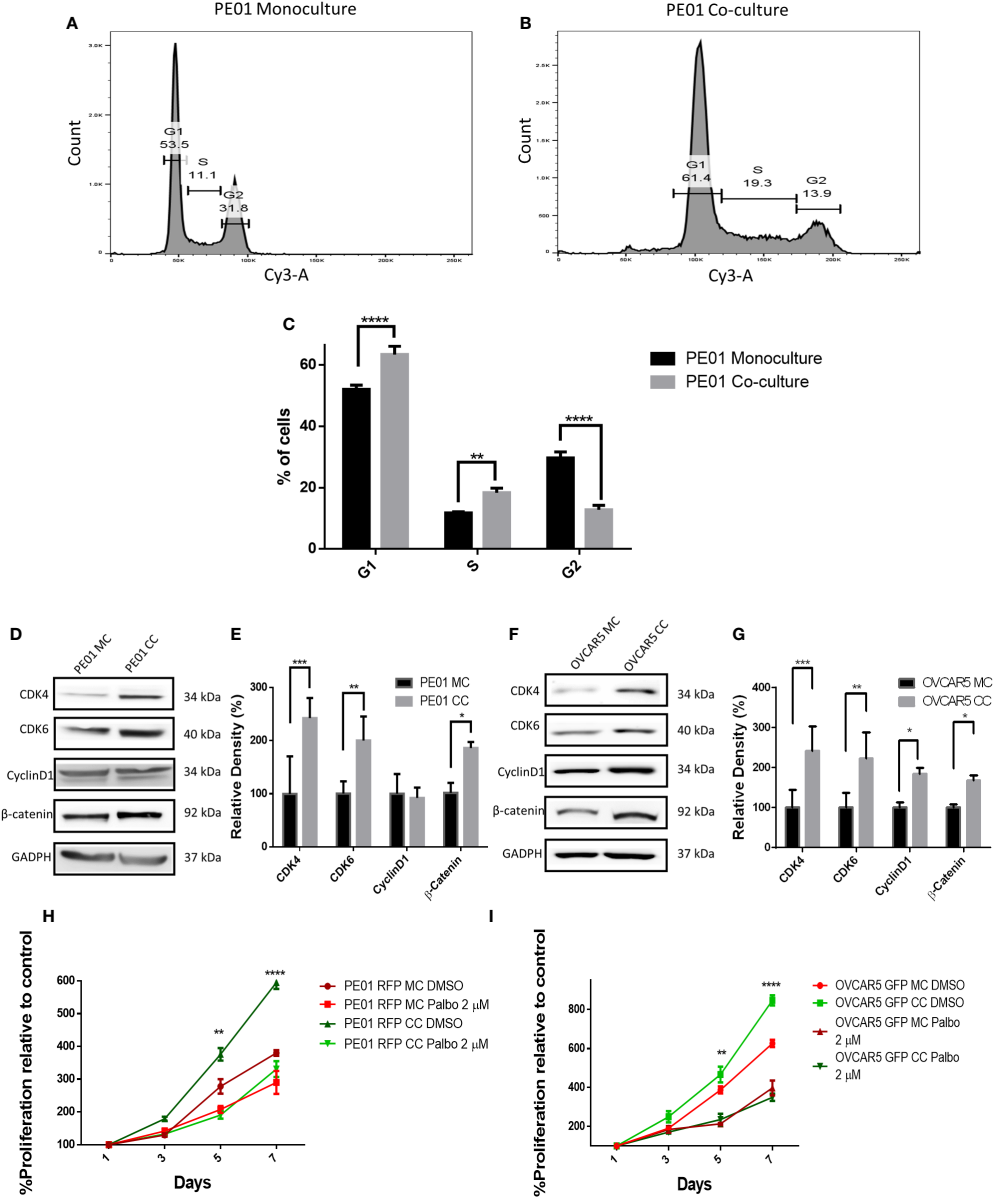
Co-culture of sensitive and resistan cells alters the proliferation rate of platinum sensitive cells. **(A-C)** Representative cell cycle analysis histograms of monocultured (MC) **(A)** and co-cultured (CC) **(B)** platinum-sensitive PE01 cells, and comparison of percentages of cells **(C)** at G1, S, and G2 phases of the cell cycle (n=3; **p<0.01; ****p<0.0001). PE01 cells were co-cultured with PE04 cells for 7 days, separated by FACS and cell cycle analysis was performed post-sorting culture. **(D-G)** Western blot analysis of cell cycle related proteins in PE01 (**D,** quantification of band density by ImageJ in **E**) and OVCAR5 cells (**F,** quantification of band density by ImageJ in **G**) after 7 days co-culture with PE04 cells and OVCAR5 CisR cells, respectively and separation by FACS. (n=3; *p<0.05 **p<0.01; ***p<0.001) **(H, I)** E ffects of 7 days treatment with palbociclib on cell proliferation (CCK8 assay) of PE01 **(H)** and OVCAR5 **(I)** cells after co-culture with PE04 and OVCAR5 CisR cells, respectively (n=3; **p<0.01; ****p<0.0001; comparing PE01 RFP CC vs. PE01 RFP CC + palbociclib 2µM and OVCAR5 GFP CC vs. OVCAR5 GFP CC + palbociclib 2µM).

To validate the transcriptomic analysis findings, cell cycle progression of PE01 cells cultured alone or co-cultured with PE04 was assessed by using PI staining and cell cycle analysis. PE01 co-cultured cells showed a significative higher percentage of cells going through G1 phase (63.36% in PE01 co-culture vs. 52.03% in PE01 monoculture p < 0.0001), significantly higher percentage of cells going through S phase (18.33% in PE01 co-culture vs. 11.7% in PE01 monoculture p < 0.01) and a significantly lower percentage of cells going through G2 phase (12.76% in PE01 co-culture vs 29.66% in PE01 monoculture, p < 0.0001, **Figures 4A-C**; **Supplementary Figure S3**), supporting that direct co-culture promotes G1-S transition of OC cells. On the other hand, cell cycle analysis of co-cultured vs mono-cultured PE04 cells showed that there were significantly fewer cells going through G1 (39.23% in PE04 co-culture vs. 54.7% in PE04 monoculture p < 0.0001) and S phase (13.46% in PE04 co-culture vs. 17.86% in PE04 monoculture p < 0.05) and a significantly higher percentage of cells in G2 phase (33.36% in PE04 co-culture vs 21.4% in monoculture, p < 0.0001, **Supplementary Figures S4A-C** and **S5**), suggesting that co-culture PE04 cells arrested in G2 phase and became senescent (15).

Given the observed differences in cell cycle progression under co-culture vs. monoculture conditions, we measured the expression levels of kinases controlling progression through G1-S; CDK4, CKD6 and Cyclin D1. Western blot analysis showed increased levels of CDK4 and CDK6 in co-cultured PE01 or OVCAR5 cells compared to monoculture cells (**Figures 4D-G**) cells, but no significant difference in cyclin D1 expression in co-cultured PE01 cells and only a modest increase in co-cultured OVCAR5 cells. β-catenin, a transcription factor known to promote cancer cell proliferation (16, 17), was increased in both co-cultured PE01 (**Figures 4D-E**) and OVCAR5 (**Figures 4F-G**) cells.

After having observed increased expression levels of CDK4/CDK6 under co-culture conditions, a CDK4/CDK6 cell cycle inhibitor was tested in PE01 or OVCAR5 cells either cultured alone or co-cultured with the corresponding platinum resistant cells (PE04 or OVCAR5-CisR). Palbociclib (at 2 µM) prevented the observed increased cell proliferation induced by co-culture with resistant cells in both PE01 and OVCAR5 cells (**Figures 4H-I**), supporting the concept that cell cycle progression is a key event dysregulated by cohabitation of sensitive and resistant cells. The inhibitor reduced cell proliferation in PE01 (IC50 = 335nM) and OVCAR5 (IC50 = 727nM) monocultured cells without reducing cell viability (**Supplementary Figures S6A, B**).

### 3.5 E2F1 regulates activation of cell cycle progression in sensitive cells after co-culture with platinum resistant cells

**Figure 5:**
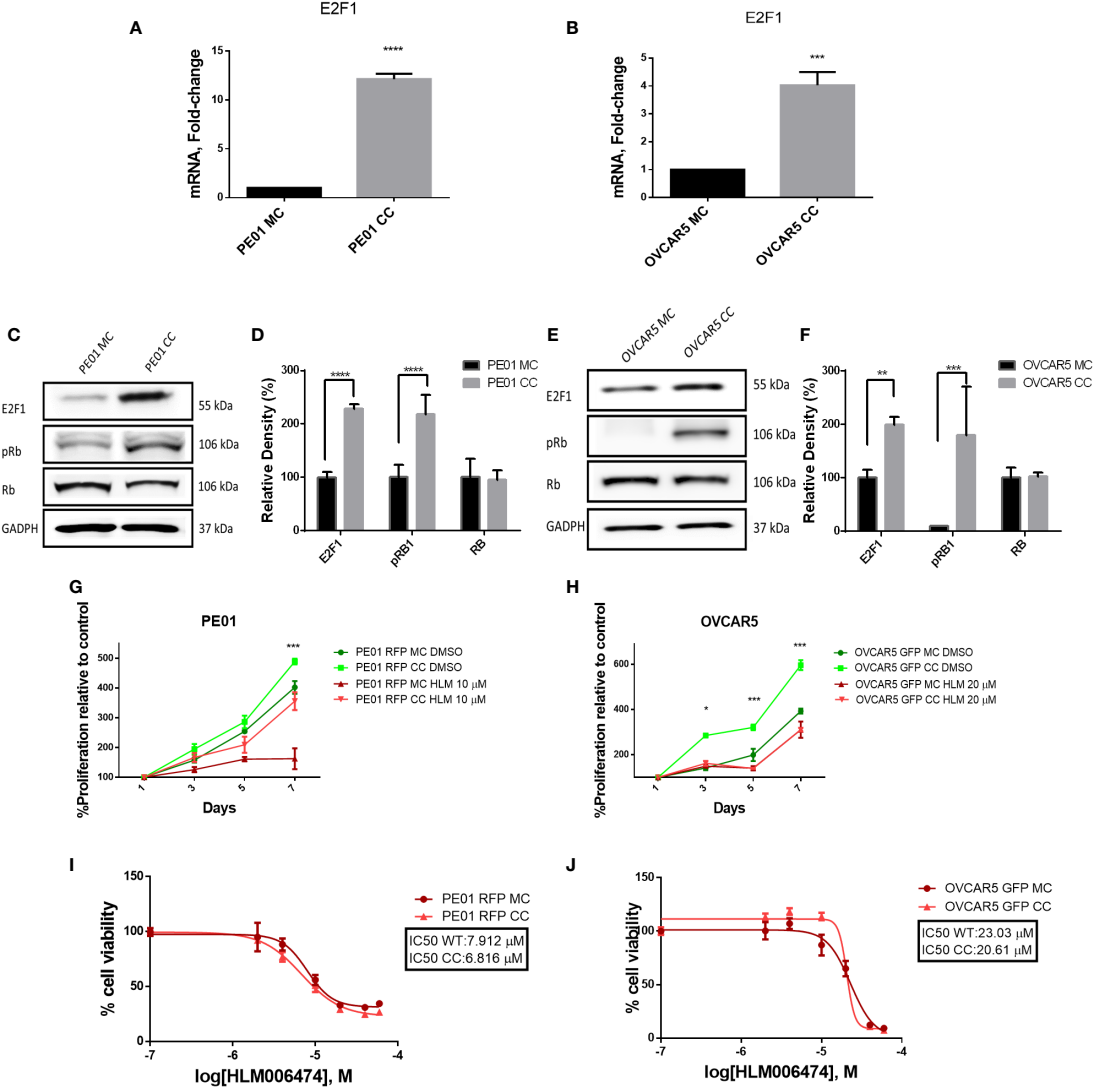
Co-culture of platinum sensitive and resistant cells induced E2F1 expression. Cells were co-cultured over 14 days and separated by FACS sorting. **(A, B)** qRT-PRC analysis of E2F1 mRNA levels in mono (MC) and co-cultured **(CC)** PE01 **(A)** and OVCAR5 **(B)** cells (n=3, p<0,05). **(C-F)** Western blot analysis of E2F1, pRb (Ser807/811) and total Rb in mono and co-cultured PE01 (**C**, quantification of band density by ImageJ in **D**) and OVCAR5 cells (**E**, quantification of band density by ImageJ in **F**; n=3; **p<0.01 ***p<0.001; ****p<0.0001) **(G, H)** Proliferation (CCK8 assay) of co-cultured cells PE01 **(G)** and OVCAR5 **(H)** treated with the E2F1 inhibitor HLM006474 (n=3; *p<0.05; ***p<0.001; comparing PE01 RFP CC vs PE01 RFP CC + HLM 10µM and OVCAR5 GFP CC vs OVCAR5 GFP CC + HLM 10µM). **(I, J)** IC_50_ for HLM006474 in mono- and co-cultured PE01 **(I)** and OVCAR5 **(J)** cells. IC_50_ was calculated based on CCK8 viability assay assessing percentage of cells surviving treatment with various concentrations of HLM006474.

As E2F1 had been found as a potential key regulator of accelerated cell cycle progression in co-cultured cells and is involved in regulation of G1-S phase transition, we measured its expression levels in PE01 and OVCAR5 cells cultured alone or co-cultured with the corresponding resistant cell line. E2F1 interacts with and is inhibited by the retinoblastoma (RB) protein, a key tumor suppressor (18). Phosphorylation of RB disrupts the complex with E2F1, allowing the transcription factor to activate the transcriptional machinery and stimulate cell proliferation (19). E2F1 was increased at mRNA levels in co-cultured cells compared to monoculture (**Figures 5A, B**). Increased E2F1 protein levels were also observed in co-cultured PE01 and OVCAR5 cells compared with cells grown in monoculture (**Figures 5C-F**). Interestingly, levels of phosphorylated RB were likewise increased in PE01 and OVCAR5 co-cultured vs. monocultured cells (**Figures 5C-F**). The increased levels of phospho-RB under co-culture conditions support the concept that E2F1 acquires increased transcriptional activity in this context.

After confirming activation of E2F1 in co-cultured vs. monocultured cells, we evaluated the effects of the selective E2F1 inhibitor HLM006474. At concentrations of 10µM for PE01 and 20µM for OVCAR5, the E2F1 inhibitor reverted the increased proliferation rate induced by co-culture (**Figures 5G, H**), supporting the concept that co-habitation of cells with different proliferation rates activate E2F1 to stimulate cell cycle progression. The inhibitor was not toxic to the cells at these concentrations, but co-cultured cells were more sensitive in comparison with monocultured cells, suggesting increased E2F1 activity under co-culture conditions (**Figures 5I, J**).

## 4 Discussion

The present study aimed to characterize the dynamic interactions between drug-sensitive and drug-resistant ovarian cancer cells in a co-culture setting and to determine the effects of these interactions on response to treatment. Our findings detect a notable increase in the proliferation rate of platinum-sensitive ovarian cancer cells when co-cultured with drug-resistant counterparts and increased response to chemotherapy. Our observations suggest the existence of a competitive mechanism within the direct co-culture microenvironment that promotes the proliferation rate of drug-sensitive cells when they are in contact with resistant cells.

Our findings are consistent with previous studies which have shown that the presence of different cellular phenotypes in proximity generates competition between cells (20, 21), leading to differential expansion of various cell subpopulations. This competition has been reported to change the phenotype of the cells – including their proliferation rates and elimination of less robust phenotypes (22–24). It has been reported that cells overexpressing specific oncogenes overtake neighboring cells that lack the gene and that this dynamic interaction between cell populations with distinct genetic make-up is important during tumor initiation. For example, overexpression of myc causes cells to acquire a “super-competitive” phenotype, leading to elimination of surrounding myc-low cells via apoptosis (25). Similarly, cells deficient in ribosomal proteins and slower proliferating, have been shown to be eliminated by neighboring faster proliferating cells in the Drosophila wing (26). This phenomenon can also be observed in the interaction between species, applicable to cohabiting plants or microbes (27, 28). Formulas of species interaction have been used to determine the dynamics of species proliferation in a co-habiting milieu.

Previous studies investigating the effects of interactions between sensitive and resistant cells, have suggested the possibility that drug resistance could be transferred from one cell to another though secreted exosomes (29). A variety of molecules contained in extracellular vesicles have been reported to be involved in the transfer of resistance features, including Annexin A6, microRNAs, or transcription factors (30–33). These studies differ from ours through their focus on indirect cell to cell communication and limited evaluation of the effects of direct cell contact and cell competition, as we do here.

We propose that the increased proliferation capability observed in drug-sensitive ovarian cancer cells when co-cultured with drug-resistant cells may be attributed to the overexpression and activity of E2F1. E2F1 is a transcription factor known to play a pivotal role in cell cycle regulation, specifically in promoting cell cycle progression from the G1 phase to the S phase (34). The increased expression of E2F1 in drug-sensitive cells under co-culture conditions suggests a potential mechanism by which these cells overcome the growth impediments posed by drug resistance. The overexpression of E2F1 and the increased proliferation potential of the sensitive cells led to reduced viability of neighboring platinum resistant cells and reduction of their number over time. These findings emphasize the potential utility of targeting E2F1 as a therapeutic avenue to mitigate the impact of drug resistance in cancer as other publications had suggested (35–41).

Our findings align with previous research that has highlighted the influence that the different cell types present in the tumor microenvironment could have upon cancer cells behavior and therapeutic responses (42, 43). The observed competitive interaction between drug-sensitive and drug-resistant cells highlights the complexity of cancer biology and underscores the importance of considering the tumor microenvironment when evaluating treatment strategies. The increased proliferation of sensitive cells recapitulates the early stages of cancer progression with fast tumor growth and generation of new metastatic foci (44–46). Furthermore, the balance between the numbers of cohabitating sensitive and resistant cells in the tumor milieu affects response to chemotherapy. The presence of small numbers of resistant cells drives the proliferation of sensitive cells and the positive response to treatment. Once chemotherapy starts, the death of drug sensitive cells opens a niche in which the drug resistant population, previously reduced by the proliferation of drug sensitive cells, can thrive.

While we have demonstrated a correlation between co-culture with drug-resistant cells, increased proliferation of drug-sensitive cells, and the role of E2F1, further mechanistic studies are needed to elucidate the precise signaling pathways and molecular interactions driving this phenomenon. Additionally, the clinical relevance of our findings warrants further investigation, as *in vivo* models and patient-derived samples may provide a more accurate representation of the tumor microenvironment and its impact on therapeutic responses.

In conclusion, our study sheds light on the intricate interplay between drug-sensitive and drug-resistant ovarian cancer cells within a co-culture setting. The observed enhancement of drug-sensitive cell proliferation and the involvement of E2F1 in stimulating the growth of this cell population provide valuable insights into potential mechanisms underlying the adaptation of cancer cells to the presence of resistant counterparts. Our research contributes to a deeper understanding of tumor biology and suggests new avenues for therapeutic interventions in ovarian cancer.

## Data availability statement

The datasets presented in this study can be found in online repositories. The names of the repository/repositories and accession number(s) can be found below: https://www.ncbi.nlm.nih.gov/geo/, GSE244626 https://uccl0-my.sharepoint.com/:f:/g/personal/aevaldiv_uc_cl/EvvGsMImEsBBsnluVeAJXU4B4S1DhCZRIlnswxcH3S9LTQ?e=nXFp8y, Raw Data.

## Author contributions

AV: Conceptualization, Data curation, Formal analysis, Investigation, Methodology, Writing – original draft, Writing – review & editing, Validation. MC: Data curation, Investigation, Methodology, Writing – review & editing. HC: Conceptualization, Investigation, Supervision, Writing – review & editing, Writing – original draft. AI: Data curation, Formal analysis, Investigation, Writing – review & editing, Validation. GZ: Formal analysis, Investigation, Writing – review & editing. HH: Formal analysis, Investigation, Writing – review & editing. DM: Conceptualization, Funding acquisition, Supervision, Writing – review & editing, Writing – original draft.
