# Supplementary material for: E2F1 mediates competition, proliferation and response to cisplatin in cohabitating resistant and sensitive ovarian cancer cells
Source: Front Oncol. 2024 Jan 26;14:1304691. doi: 10.3389/fonc.2024.1304691 (PMC10853425; doi:10.3389/fonc.2024.1304691)
Supplement: Supplementary file 1 [file DataSheet_1.docx]

**Supplemental Material**

# Data Availability

To review GEO accession GSE244626 go to: <https://urldefense.com/v3/__https://www.ncbi.nlm.nih.gov/geo/query/acc.cgi?acc=GSE244626__;!!Dq0X2DkFhyF93HkjWTBQKhk!S7JXvIR99Ep7IAd8mYBGT_mdVzvtjRGnZ278iazK_0HHThvfNMRqNviDkhlnSXRRYnsPIVz340hFOIQ58HJ7ZxbVY7B7$>

The following secure token has been created to allow review of record GSE244626 while it remains in private status: **gdunwiicxnyjpkr**

To review Raw Data presented in this manuscript, go to:

<https://uccl0-my.sharepoint.com/:f:/g/personal/aevaldiv_uc_cl/EvvGsMImEsBBsnluVeAJXU4B4S1DhCZRIlnswxcH3S9LTQ?e=nXFp8y>

The following password needs to be used: **gdunwiicxnyjpkr**


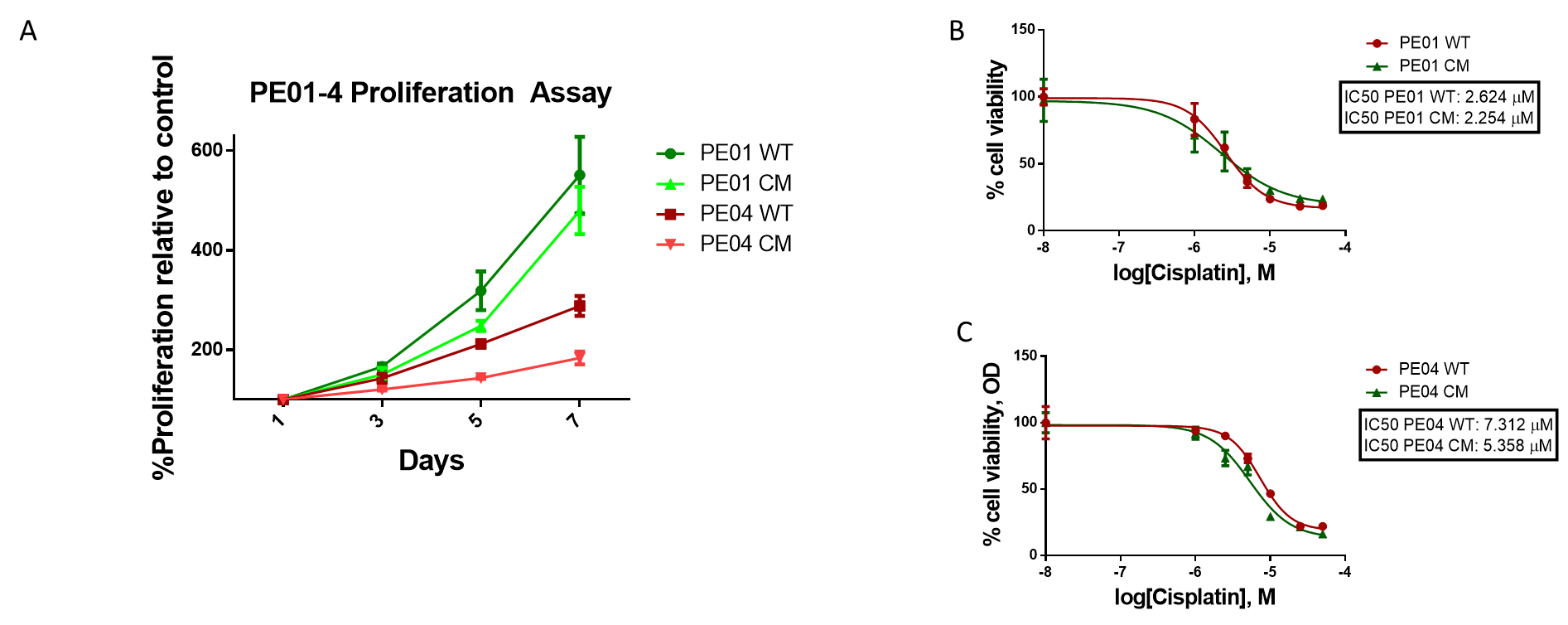


**Supplemental Figure S1:** **Conditioned media of PE01/PE04 does not affect cell proliferation or the cellular responses to cisplatin. (A)** Effects of conditioned media on the proliferation of PE01 and PE04 cells. The cells were cultured in regular or conditioned (CM) media from the other cell line for 7 days. The proliferation rates were measured by using CCK8 assay. **(B, C)** IC50 values for cisplatin in PE01 **(B)** and PE04 **(C)** cells cultured in regular or conditioned media form the other cell line. Cells were treated for 24 hrs and viable cells were assessed by using the CCK8 assay on day 4, after a 72 hrs recovery period.


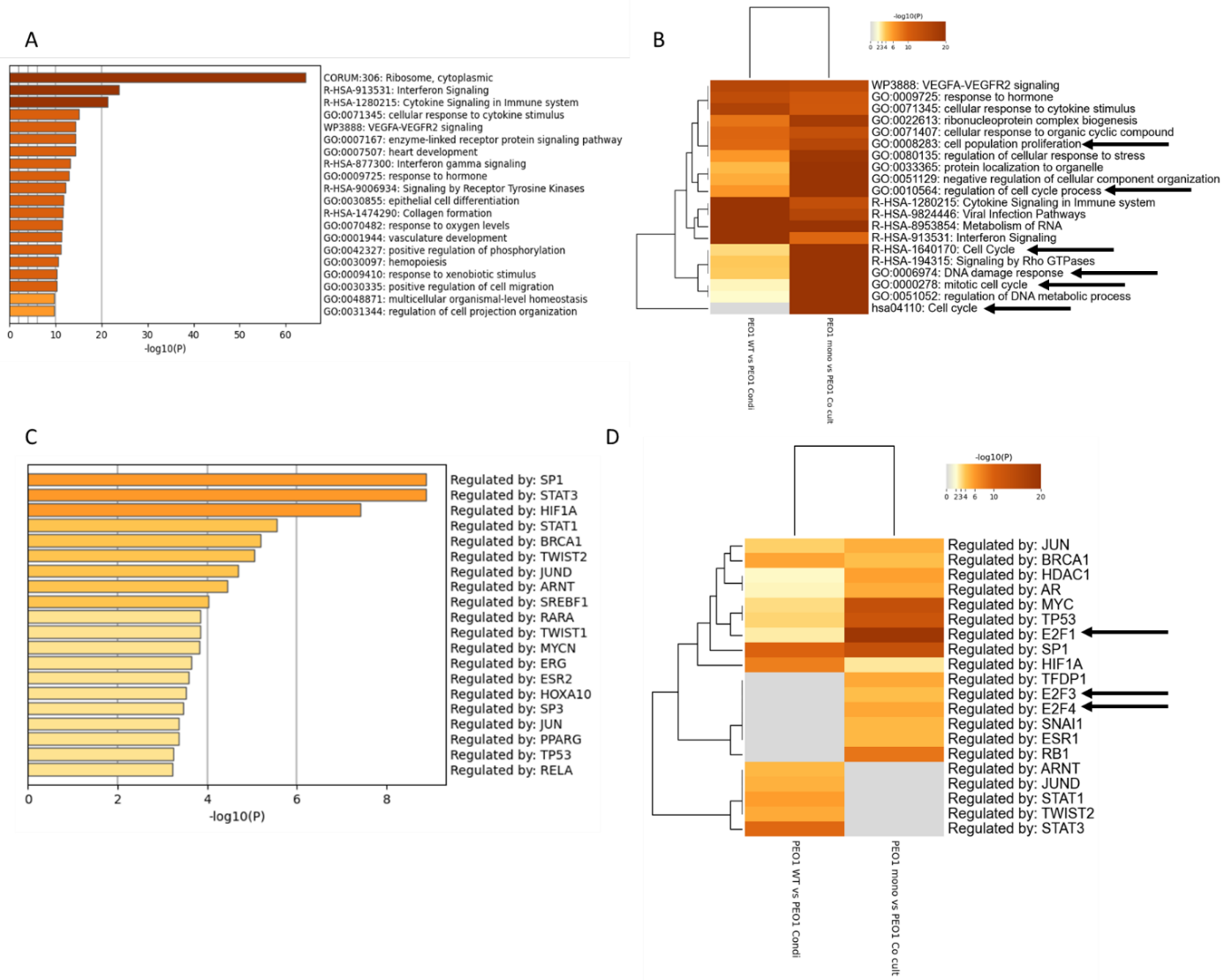


**Supplemental Figure S2. RNA-Seq analysis comparing the DEGs and enriched pathways of PE01 cells cultured in conditioned media of PE04 cells vs PE01 co-cultured with PE04 cells.** RNA-Seq analysis was performed using the web-based Metascape portal. **(A)** Pathway Enrichment analysis of DEGs (FDR<0.05) identified in PE01 cells cultured in conditioned media from PE04 cells vs regular media. **(B)** Pathways involved in the regulation of cell cycle are enriched in co-cultured cells (Black Arrows). Pathway Enrichment Analysis was performed to compare the enriched pathways identified in conditioned media group vs co-cultured cells. **(C)** The main effectors identified when cells were treated with conditioned media. **(D)** E2F gene family is a main effector in co-cultured cells (Black arrows). The main effector analysis compared the enriched pathways identified in conditioned media group vs co-culture group.


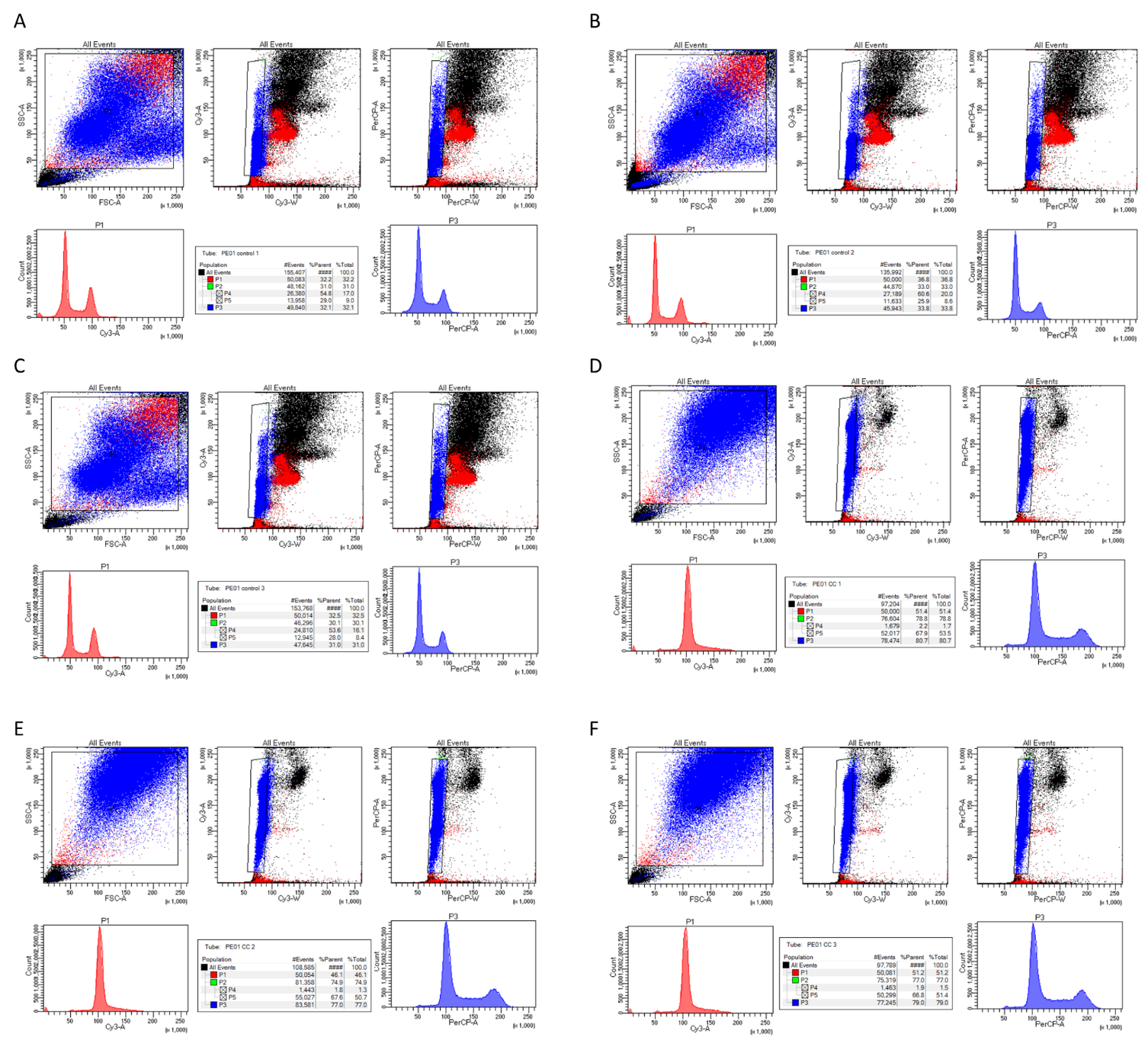


**Supplemental Figure S3. Gating for cell cycle analysis of PE01 cells.** Raw data and settings of cell cycle analysis presented in Figures 4A-C. **(A-C)** 3 replicates of PE01 monoculture **(D-F)** 3 replicates of PE01 co-cultured with PE04 for 3 days before FACS sorting.


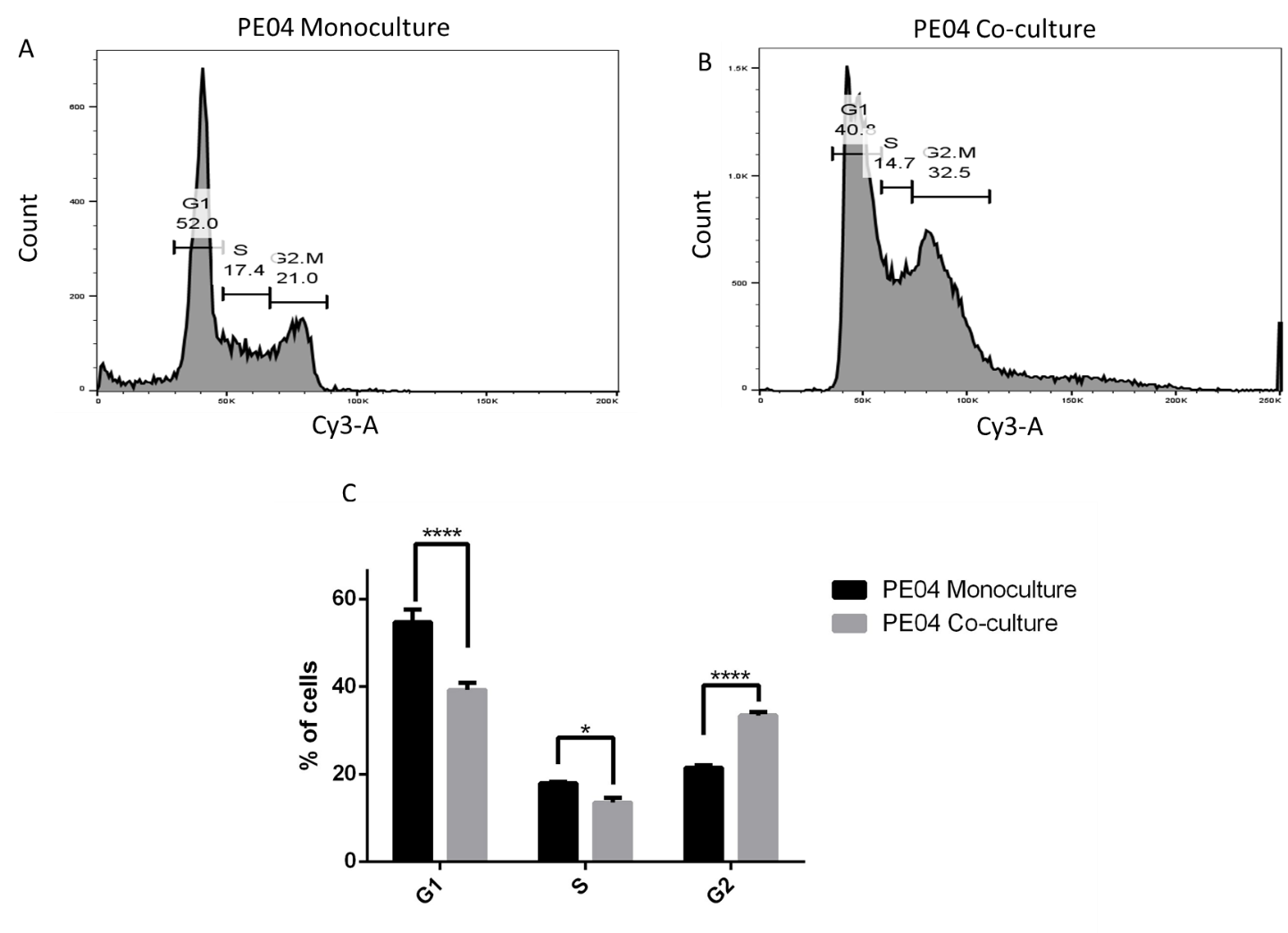


**Supplemental Figure S4**: Cell cycle analysis of co-cultured and monocultured PE04 cells. (**A-C**) Representative cell cycle analysis histograms of monocultured (**A**) and co-cultured (**B**) platinum-resistant PE04 cells and comparison of percentages of cells (**C)** in G1, S, and G2 phases of the cell cycle (n=3; *p<0.05; ****p<0.0001).


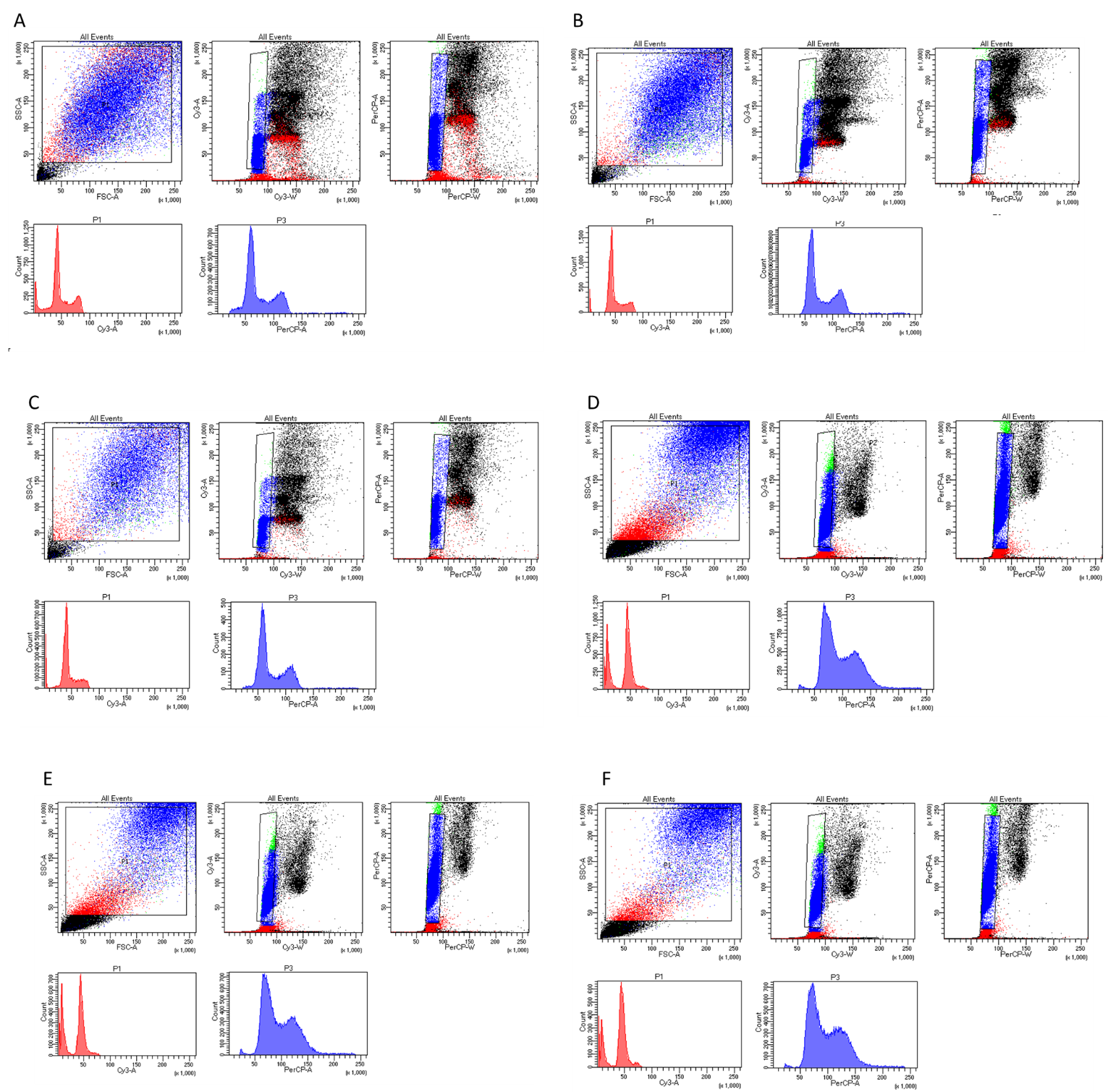


**Supplemental Figure S5. Gating settings for cell cycle analysis of PE04 cells.** Raw data and settings of cell cycle analysis presented in Supp Figure S4A-C: **(A-C)** 3 replicates of PE04 monoculture **(D-F)** 3 replicates of PE04 co-cultured with PE01 for 3 days before FACS sorting.


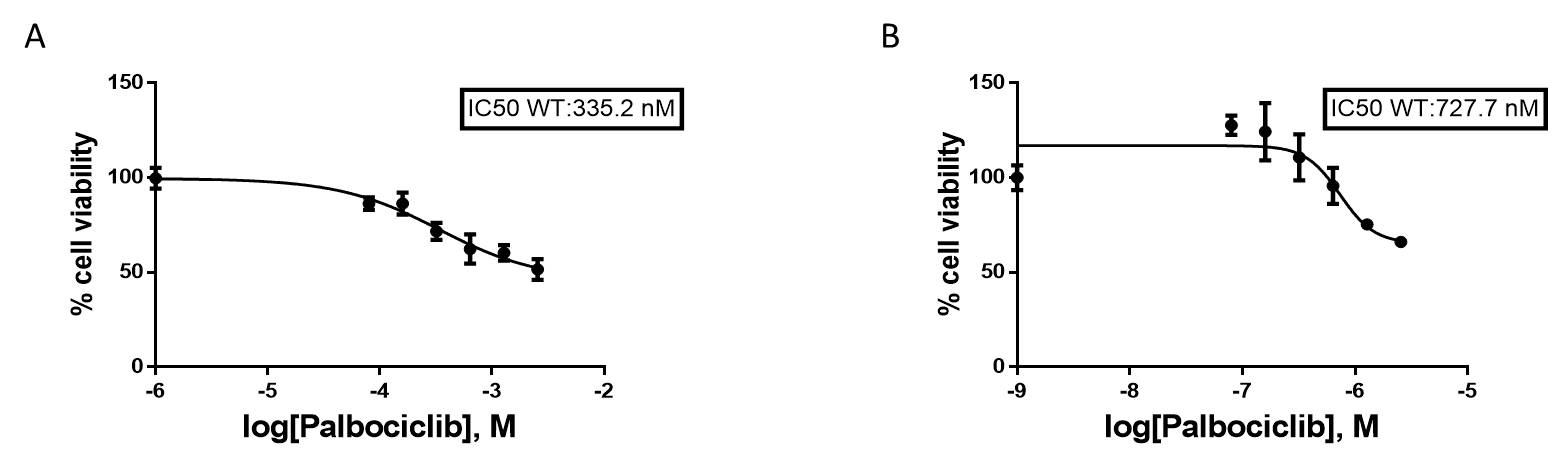


**Supplemental Figure S6. Treatment with palbociclib does not affect cell viability.** Effects of palbociclib on viability of PE01 and OVCAR5 cells measured as relative cell viability and IC_50_. PE01 **(A)** and OVCAR5 **(B)** cells were treated with a dose range of palbociclib concentrations for 7 days and viable cells were measured by using CCK8 assay.

**Supp Table S1**: Media utilized for the culture of cells.

| **Cell line** | **Cell Culture Media** |
| --- | --- |
| **OVCAR5 and OVCAR5 CisR** | RPMI 1640 (Corning), 10% fetal bovine serum (FBS) (Corning), 1% penicillin/streptomycin (Sigma), and 1X GlutaMAX (Gibco). |
| **PE01 and PE04** | RPMI 1640 (Corning), 10% fetal bovine serum (FBS) (Corning), 1% penicillin/streptomycin (Sigma), 2mM GlutaMAX (Gibco) and 2mM Sodium Pyruvate (Gibco) |

**Supp Table S2**: List of primary antibodies.

| **Antibodies** | **Source** | **Cat#** |
| --- | --- | --- |
| **E2F1 Monoclonal Antibody (KH95)** | Fisher/ Invitrogen | 32-1400 |
| **Human RB1 Antibody** | R&D Systems | MAB6495 |
| **Phospho-Rb (Ser807/811) (D20B12) XP® Rabbit mAb #8516T** | Cell Signaling Technology | 8516S |
| **CDK4** | Cell Signaling Technology | DCS156 |
| **CDK6** | Cell Signaling Technology | DCS83 |
| **CyclinD1** | Cell Signaling Technology | DCS6 |
| **Β-Catenin** | ECM Biosciences | CM1181 |
| **Anti-GAPDH** | Meridian | H86045M |

**Supp Table S3** List of Primers for qPCR.

| **Gene** | **Forward Primer (5’ to 3’)** | **Reverse Primer (5’ to 3’)** |
| --- | --- | --- |
| **E2F1** | TGGACCTGGAAACTGACCAT | ATAGCGTGACTTCTCCCCCG |
| **GAPDH** | GATTCCACCCATGGCAAATTCC | CACGTTGGCAGTGGGGAC |

**Supp Table S4:** TOP 100 DEGs of PE01 cells co-cultured with PE04 vs monoculture.

| Hgnc symbol | Fold Change | p-Value | FDR |
| --- | --- | --- | --- |
| ABCG2 | 10.2736 | 3.95E-10 | 2.03E-08 |
| DMBT1 | 7.851154 | 9.71E-15 | 1.56E-12 |
| AKR1D1 | 6.428288 | 2.47E-08 | 7.48E-07 |
| APLN | 6.1659 | 2.65E-22 | 2.00E-19 |
| CFAP44 | 6.07254 | 4.63E-14 | 6.34E-12 |
| KIF6 | 5.935293 | 1.11E-08 | 3.77E-07 |
| ADRA1B | 5.885196 | 1.83E-19 | 8.63E-17 |
| SUSD5 | 5.181814 | 3.15E-24 | 3.17E-21 |
| SEMA3D | 4.72604 | 2.52E-14 | 3.69E-12 |
| FOSB | 4.504352 | 1.44E-13 | 1.84E-11 |
| FSIP1 | 4.441065 | 4.07E-06 | 6.25E-05 |
| ESCO2 | 4.379228 | 2.14E-12 | 1.97E-10 |
| NOG | 4.232929 | 3.87E-16 | 8.45E-14 |
| LIPG | 4.058452 | 2.77E-14 | 4.01E-12 |
| SOAT2 | 3.992765 | 1.34E-12 | 1.37E-10 |
| EIF5AL1 | 3.988693 | 0.000279 | 0.002143 |
| HIST2H2AC | 3.828651 | 5.85E-05 | 0.000578 |
| RRM2 | 3.807022 | 1.06E-26 | 1.59E-23 |
| HNRNPM | 3.710929 | 1.04E-17 | 3.27E-15 |
| CACNA1D | 3.53688 | 3.92E-05 | 0.000418 |
| TXK | 3.519151 | 4.65E-05 | 0.000476 |
| CPA2 | 3.482873 | 2.69E-05 | 0.000306 |
| MMP16 | 3.38717 | 0.000104 | 0.000943 |
| RCBTB2 | 3.367192 | 4.25E-08 | 1.20E-06 |
| CCL2 | 3.360934 | 0.000122 | 0.00107 |
| FAM111B | 3.343285 | 4.72E-20 | 2.54E-17 |
| PDE4D | 3.3237 | 4.90E-09 | 1.86E-07 |
| IVL | 3.249932 | 8.79E-06 | 0.000118 |
| ST6GAL2 | 3.216235 | 1.13E-10 | 6.46E-09 |
| KCNC1 | 3.201655 | 5.69E-09 | 2.12E-07 |
| FAM222A | 3.185211 | 3.58E-10 | 1.86E-08 |
| RARRES1 | 3.158891 | 1.39E-09 | 6.14E-08 |
| AMH | 3.142634 | 1.04E-05 | 0.000137 |
| CIP2A | 3.043487 | 1.42E-08 | 4.66E-07 |
| MPHOSPH6 | 3.036645 | 2.41E-06 | 4.03E-05 |
| ILDR2 | 3.01849 | 2.86E-06 | 4.64E-05 |
| DHFR | 3.009875 | 4.82E-11 | 3.26E-09 |
| SERPINB8 | 3.003801 | 2.13E-15 | 3.86E-13 |
| POLA1 | 2.962704 | 2.78E-18 | 1.05E-15 |
| COL12A1 | 2.904864 | 1.69E-12 | 1.64E-10 |
| WDR77 | 2.89742 | 3.29E-10 | 1.74E-08 |
| MPDU1 | 2.877826 | 6.06E-09 | 2.25E-07 |
| SPC25 | 2.861806 | 4.33E-14 | 6.10E-12 |
| HSPH1 | 2.838781 | 7.78E-18 | 2.55E-15 |
| C1orf112 | 2.825407 | 3.34E-07 | 7.38E-06 |
| FBXL7 | 2.817813 | 0.000136 | 0.001176 |
| DTWD2 | 2.813574 | 1.33E-06 | 2.42E-05 |
| BMPER | 2.803538 | 3.91E-05 | 0.000418 |
| CHAC2 | 2.792482 | 9.83E-10 | 4.54E-08 |
| ITGB1BP2 | 2.792184 | 2.26E-06 | 3.81E-05 |
| MAMLD1 | 2.791926 | 1.29E-09 | 5.77E-08 |
| ZBED6CL | 2.776095 | 1.31E-14 | 1.98E-12 |
| CENPE | 2.74315 | 4.91E-07 | 1.03E-05 |
| OAS3 | 2.740875 | 2.39E-16 | 5.45E-14 |
| STXBP4 | 2.736283 | 1.52E-09 | 6.64E-08 |
| DNAJA1 | 2.730681 | 2.50E-17 | 7.39E-15 |
| TEX261 | 2.729921 | 8.72E-16 | 1.75E-13 |
| INTS7 | 2.728757 | 2.19E-06 | 3.71E-05 |
| CAP2 | 2.725062 | 1.24E-06 | 2.29E-05 |
| DOK3 | 2.702434 | 2.21E-09 | 9.09E-08 |
| NCAPG | 2.694267 | 6.72E-10 | 3.29E-08 |
| KCNB2 | 2.692478 | 0.001536 | 0.008431 |
| PDK1 | 2.663379 | 6.09E-09 | 2.25E-07 |
| MYBL2 | 2.653625 | 1.94E-06 | 3.36E-05 |
| HSP90AA1 | 2.648636 | 1.45E-14 | 2.17E-12 |
| MCM10 | 2.648589 | 4.60E-14 | 6.34E-12 |
| ZWILCH | 2.62536 | 9.53E-06 | 0.000126 |
| WDR17 | 2.620103 | 3.51E-08 | 1.02E-06 |
| HAS3 | 2.619139 | 7.04E-10 | 3.43E-08 |
| ADGB | 2.606092 | 1.22E-08 | 4.07E-07 |
| PDE4B | 2.605302 | 2.74E-12 | 2.45E-10 |
| C12orf4 | 2.598393 | 1.87E-07 | 4.48E-06 |
| ERCC6L | 2.598011 | 1.52E-12 | 1.50E-10 |
| KBTBD8 | 2.593095 | 2.09E-08 | 6.54E-07 |
| MBNL3 | 2.578895 | 9.73E-08 | 2.50E-06 |
| DHX9 | 2.555702 | 1.85E-14 | 2.74E-12 |
| SRSF1 | 2.539168 | 7.98E-06 | 0.000109 |
| SLC25A12 | 2.535467 | 1.06E-07 | 2.70E-06 |
| TMEM19 | 2.527339 | 9.34E-07 | 1.79E-05 |
| SDC1 | 2.525819 | 1.83E-12 | 1.76E-10 |
| MNS1 | 2.52304 | 7.32E-11 | 4.53E-09 |
| SSX2IP | 2.522831 | 3.02E-07 | 6.77E-06 |
| SLC12A2 | 2.499343 | 7.97E-11 | 4.86E-09 |
| ID3 | 2.496661 | 3.30E-13 | 3.89E-11 |
| PARM1 | 2.484968 | 4.98E-07 | 1.04E-05 |
| DKK1 | 2.462586 | 6.37E-07 | 1.29E-05 |
| ROR1 | 2.461608 | 1.34E-10 | 7.63E-09 |
| CCNE2 | 2.459778 | 8.40E-09 | 2.93E-07 |
| SPAG1 | 2.457526 | 8.46E-12 | 6.75E-10 |
| DDIAS | 2.454604 | 0.000127 | 0.001109 |
| SPDL1 | 2.450168 | 7.88E-13 | 8.48E-11 |
| EGR1 | 2.443281 | 3.22E-07 | 7.17E-06 |
| MMS22L | 2.442839 | 0.000268 | 0.002081 |
| SAPCD2 | 2.440132 | 1.28E-12 | 1.33E-10 |
| KIF15 | 2.438872 | 1.77E-09 | 7.52E-08 |
| HSPA4L | 2.437 | 1.55E-06 | 2.75E-05 |
| DUSP1 | 2.433862 | 7.74E-10 | 3.71E-08 |
| IFI6 | 2.429463 | 2.89E-07 | 6.50E-06 |
| HAS2 | 2.429461 | 1.10E-05 | 0.000143 |
| ENC1 | 2.429441 | 1.91E-09 | 8.03E-08 |

**Supp Table S5:** TOP 100 DEGs of PE01 cells treated with conditioned media of PE04 vs regular media.

| Hgnc symbol | Fold Change | p-Value | FDR |
| --- | --- | --- | --- |
| IFI6 | 9.652273 | 1.1E-226 | 1.6E-222 |
| OAS2 | 6.865429 | 4.74E-39 | 5.56E-36 |
| MX1 | 6.724344 | 1E-213 | 7.8E-210 |
| IFI44L | 6.374697 | 1.9E-132 | 7.2E-129 |
| XAF1 | 4.658295 | 3.33E-23 | 2.31E-20 |
| PPP1R1A | 4.064766 | 3.64E-10 | 6.45E-08 |
| TSHZ2 | 3.917105 | 1.17E-15 | 4.16E-13 |
| IFI27 | 3.861133 | 7E-159 | 3.5E-155 |
| XKR4 | 3.659112 | 1.25E-09 | 1.91E-07 |
| BAALC | 3.625362 | 2.26E-07 | 2.14E-05 |
| OAS1 | 3.445041 | 1.26E-44 | 1.61E-41 |
| BST2 | 3.442068 | 1.25E-30 | 1.12E-27 |
| IFITM1 | 3.267771 | 9.21E-36 | 1.00E-32 |
| COL6A2 | 3.253183 | 2.08E-05 | 0.00091 |
| NCAM1 | 3.184955 | 7.26E-06 | 0.000392 |
| OAS3 | 3.064591 | 3.32E-90 | 1.01E-86 |
| SLC2A9 | 2.976812 | 1.03E-12 | 2.63E-10 |
| ESM1 | 2.889535 | 1.34E-09 | 2.03E-07 |
| IFI44 | 2.730862 | 2.44E-49 | 3.73E-46 |
| IFIT1 | 2.70077 | 2.65E-69 | 6.73E-66 |
| CCBE1 | 2.684661 | 1.90E-66 | 4.15E-63 |
| KRT4 | 2.550868 | 9.77E-65 | 1.86E-61 |
| A2ML1 | 2.334102 | 0.000987 | 0.017141 |
| FAM196B | 2.240301 | 6.23E-07 | 5.28E-05 |
| KRT3 | 2.210009 | 8.64E-05 | 0.002718 |
| IRF7 | 2.183251 | 6.41E-21 | 4.08E-18 |
| STAT1 | 2.179068 | 1.44E-57 | 2.43E-54 |
| SLC35F1 | 2.164634 | 1.10E-06 | 8.44E-05 |
| ADGRF5 | 2.141147 | 0.000692 | 0.013316 |
| ZNF560 | 2.131248 | 0.002219 | 0.030845 |
| SKAP1 | 2.111961 | 0.000263 | 0.006438 |
| IRF9 | 2.082211 | 2.00E-13 | 5.65E-11 |
| PCDH19 | 2.07708 | 5.81E-05 | 0.001968 |
| MRAP2 | 2.071349 | 0.002445 | 0.033174 |
| PNOC | 2.069553 | 0.000327 | 0.007656 |
| THSD7B | 2.036217 | 0.003319 | 0.041052 |
| PLSCR1 | 1.999321 | 6.14E-45 | 8.51E-42 |
| RP1 | 1.995458 | 0.000333 | 0.007747 |
| FOLH1 | 1.987726 | 0.002421 | 0.032901 |
| TMEM74 | 1.973355 | 0.000378 | 0.008472 |
| SIAH3 | 1.966812 | 1.70E-07 | 1.66E-05 |
| SP110 | 1.963256 | 1.72E-18 | 7.94E-16 |
| ZMYND10 | 1.960441 | 0.000743 | 0.013987 |
| DOK3 | 1.960376 | 4.23E-05 | 0.001556 |
| RAI2 | 1.942986 | 4.48E-05 | 0.001614 |
| NOG | 1.937292 | 0.000111 | 0.003279 |
| WNK3 | 1.914833 | 0.000893 | 0.015934 |
| MXRA5 | 1.914342 | 1.79E-16 | 7.19E-14 |
| KLHL14 | 1.903619 | 1.32E-16 | 5.45E-14 |
| ZNF208 | 1.89712 | 0.003453 | 0.042156 |
| PAK3 | 1.892994 | 0.000438 | 0.009577 |
| PRKG1 | 1.886268 | 6.76E-05 | 0.002231 |
| VEPH1 | 1.879961 | 1.23E-17 | 5.50E-15 |
| PARP9 | 1.872375 | 6.49E-34 | 6.60E-31 |
| CWH43 | 1.870579 | 1.54E-05 | 0.00073 |
| CBR1 | 1.859637 | 0.000275 | 0.006669 |
| PCOLCE2 | 1.852865 | 0.00202 | 0.028871 |
| SYT11 | 1.852401 | 0.001909 | 0.027722 |
| REEP1 | 1.851964 | 0.001941 | 0.028031 |
| VAV3 | 1.838704 | 8.46E-10 | 1.39E-07 |
| SAMD9 | 1.836626 | 1.96E-33 | 1.87E-30 |
| KRT78 | 1.830399 | 0.000341 | 0.007829 |
| ANKRD2 | 1.82955 | 6.78E-14 | 2.11E-11 |
| EDNRA | 1.827675 | 3.05E-05 | 0.001237 |
| ID1 | 1.821633 | 3.02E-30 | 2.56E-27 |
| GIPC2 | 1.818977 | 3.27E-09 | 4.61E-07 |
| ADRB2 | 1.815992 | 0.000146 | 0.00412 |
| CADPS | 1.812375 | 2.09E-07 | 2.01E-05 |
| MCC | 1.78193 | 7.15E-12 | 1.56E-09 |
| FGF1 | 1.780704 | 0.002579 | 0.034586 |
| PLCB1 | 1.769674 | 4.53E-08 | 5.00E-06 |
| PCSK5 | 1.765237 | 5.76E-19 | 2.75E-16 |
| ASTN1 | 1.763849 | 6.29E-05 | 0.002114 |
| BDNF | 1.73673 | 8.70E-17 | 3.68E-14 |
| DDX60 | 1.73033 | 1.08E-19 | 5.68E-17 |
| PARP14 | 1.730158 | 9.17E-30 | 6.99E-27 |
| SLC8A1 | 1.725472 | 1.23E-06 | 9.23E-05 |
| HERC6 | 1.723178 | 8.24E-16 | 2.99E-13 |
| PPM1K | 1.722691 | 0.000211 | 0.005482 |
| ANGPTL4 | 1.712837 | 5.96E-16 | 2.22E-13 |
| SYTL2 | 1.703709 | 3.31E-20 | 1.94E-17 |
| TRABD2A | 1.696723 | 1.91E-15 | 6.60E-13 |
| ADRA1B | 1.689451 | 2.24E-05 | 0.000961 |
| NPFFR1 | 1.680631 | 0.004001 | 0.046652 |
| SYT16 | 1.669159 | 4.89E-08 | 5.37E-06 |
| HHIPL2 | 1.667205 | 0.000592 | 0.012025 |
| NRXN3 | 1.65893 | 1.10E-06 | 8.44E-05 |
| IVL | 1.652253 | 6.89E-05 | 0.002266 |
| GPAT3 | 1.633239 | 4.63E-05 | 0.001644 |
| IL1R2 | 1.627907 | 0.000803 | 0.014756 |
| KCNH3 | 1.621541 | 9.82E-07 | 7.68E-05 |
| NCCRP1 | 1.618185 | 0.000605 | 0.012165 |
| UPK2 | 1.614451 | 0.004227 | 0.048688 |
| CLGN | 1.608649 | 0.000522 | 0.010971 |
| APLN | 1.593968 | 0.000869 | 0.01565 |
| DTX3L | 1.592926 | 1.70E-21 | 1.12E-18 |
| B3GAT1 | 1.584327 | 0.000718 | 0.013656 |
| CCNYL1 | 1.583768 | 8.34E-13 | 2.19E-10 |
| SPTLC3 | 1.582753 | 0.000102 | 0.003083 |
| ADGRG2 | 1.580583 | 3.29E-07 | 2.99E-05 |
